# Composition of Lignocellulose Hydrolysate in Different Biorefinery Strategies: Nutrients and Inhibitors

**DOI:** 10.3390/molecules29102275

**Published:** 2024-05-11

**Authors:** Yilan Wang, Yuedong Zhang, Qiu Cui, Yingang Feng, Jinsong Xuan

**Affiliations:** 1 Department of Bioscience and Bioengineering, School of Chemistry and Biological Engineering, University of Science and Technology Beijing, 30 Xueyuan Road, Beijing 100083, China; 2 CAS Key Laboratory of Biofuels, Shandong Provincial Key Laboratory of Synthetic Biology, Shandong Engineering Laboratory of Single Cell Oil, Qingdao Institute of Bioenergy and Bioprocess Technology, Chinese Academy of Sciences, 189 Songling Road, Qingdao 266101, China; 3 Shandong Energy Institute, 189 Songling Road, Qingdao 266101, China; 4 Qingdao New Energy Shandong Laboratory, 189 Songling Road, Qingdao 266101, China; 5 State Key Laboratory of Microbial Technology, Shandong University, Qingdao 266237, China; 6 University of Chinese Academy of Sciences, Beijing 100049, China

**Keywords:** lignocellulose, cellulase, biorefinery, hydrolysate, fermentable sugar, fermentation inhibitor, pretreatment, saccharification, cellulosome

## Abstract

The hydrolysis and biotransformation of lignocellulose, i.e., biorefinery, can provide human beings with biofuels, bio-based chemicals, and materials, and is an important technology to solve the fossil energy crisis and promote global sustainable development. Biorefinery involves steps such as pretreatment, saccharification, and fermentation, and researchers have developed a variety of biorefinery strategies to optimize the process and reduce process costs in recent years. Lignocellulosic hydrolysates are platforms that connect the saccharification process and downstream fermentation. The hydrolysate composition is closely related to biomass raw materials, the pretreatment process, and the choice of biorefining strategies, and provides not only nutrients but also possible inhibitors for downstream fermentation. In this review, we summarized the effects of each stage of lignocellulosic biorefinery on nutrients and possible inhibitors, analyzed the huge differences in nutrient retention and inhibitor generation among various biorefinery strategies, and emphasized that all steps in lignocellulose biorefinery need to be considered comprehensively to achieve maximum nutrient retention and optimal control of inhibitors at low cost, to provide a reference for the development of biomass energy and chemicals.

## 1. Introduction

Lignocellulosic biomass (LCB), as one of the most abundant renewable resources in the world, plays an increasingly important role in the circular economy and sustainable development, thus attracting great attention in various research areas. These studies are dedicated to developing techniques in the bioconversion process known as biorefinery which converts lignocellulosic substrates into products such as biofuels, bioplastics, bio-based chemicals, and bio-gases, to substitute non-renewable fossil-based fuels partially or completely [1,2,3,4]. The biorefinery processes of lignocellulosic biomass can be classified into two groups based on conversion approaches and intermediate products (platform molecules). One is the thermochemical conversion process involving pyrolysis which converts biomass into hydrogen and carbon monoxide (the syngas platform) and downstream chemical conversion or biological fermentation which synthesizes various downstream products [5]. The other is the biochemical or biological process that converts lignocellulosic biomass into sugars (the sugar platform) for subsequent conversion. Many studies and recent advances in thermochemical conversion processes have been reported and summarized [6,7,8,9]. In this review article, we will mainly focus on the strategies related to lignocellulosic biorefinery via the sugar platform.

**Figure 1:**
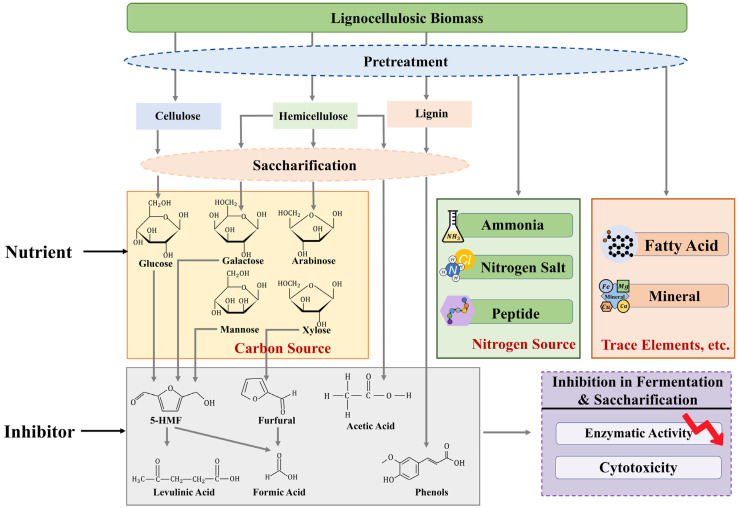
Nutrients and inhibitors present in lignocellulosic hydrolysate.

Cellulose and hemicellulose are major polysaccharides in lignocellulose. In the process via the sugar platform, these carbohydrate polymers are enzymatically hydrolyzed and release fermentable sugars such as monosaccharides or oligosaccharides, generating lignocellulosic hydrolysate (sugar solution), which can be further utilized by microbes [10]. Lignocellulosic biomass is a tightly interwoven matrix, and pretreatment is a required step for breaking this highly complex structure with a recalcitrant nature by various chemical, physical, physicochemical, and biological methods [11]. Then, the saccharification of cellulose and hemicellulose polymers is achieved by enzymatic hydrolysis with cellulases and hemicellulases [12,13]. In addition to cellulose and hemicellulose, lignin is also one major component of lignocellulose, as a network phenolic polymer providing structural reinforcement and resilience [14]. Moreover, other substances such as small amounts of pectins, cutins, waxes, lipids, tannins, terpenes, alkaloids, and resins are also found in lignocellulosic biomass, and they vary significantly with the species [15,16]. These structural constitutes can be retained in the lignocellulosic hydrolysate with varying degrees in different biorefinery strategies and processes. Most pretreatment processes are accompanied by complex physical and chemical changes, and all lignocellulosic compositions may undergo chemical reactions and produce new compounds that are potentially present in the lignocellulosic hydrolysate [17,18,19]. Therefore, the chemical composition of lignocellulosic hydrolysate is often complex and heterogeneous (Figure 1).

Lignocellulosic hydrolysate is typically used for further microbial fermentation to produce the final biofuels and bio-based chemicals, but the relatively low price of the end products makes it economically impractical to separate and purify fermentable sugars from lignocellulosic hydrolysate. Therefore, it is crucial that we understand the various chemical compositions (including the nutrients and inhibitors) of the lignocellulosic hydrolysate and their impact on the subsequent fermentation step in depth for the development of economically feasible biorefinery strategies.

In the downstream fermentation process of biorefinery, the nutritional requirements for fermenting microbes include carbon sources, nitrogen sources, trace elements, and other essential nutrients. In most studies, lignocellulosic hydrolysates are used as carbon sources, and whether hexoses (such as glucose) or pentoses (such as xylose) in the hydrolysates can be fully utilized by microbes or not will be taken into account [20] and determine the sugar conversion rate. In fact, due to the complex structural components of lignocellulosic materials themselves and the complexity of the pretreatment and saccharification processes, not only carbon sources but also many other complex components are contained in lignocellulosic hydrolysates. For example, nitrogen sources are necessary for microbial growth and often present in the lignocellulosic hydrolysate. The raw materials themselves, especially those agricultural wastes, naturally contain certain amounts of nitrogen, such as proteins, amino acids, and other nitrogen compounds [15,16,21]. These nitrogen compounds may be retained during certain pretreatment and saccharification processes and, ultimately, remain in lignocellulosic hydrolysates. Furthermore, if ammonia or nitrates are added during the pretreatment or saccharification in some process designs, they may also remain in the lignocellulosic hydrolysates with higher concentrations than the amount of nitrogen possibly required for downstream fermentation [22,23]. Other minor but essential nutrients such as phosphorus, calcium, magnesium, and iron also have significant impacts on growth and production, and all their contents in the lignocellulosic hydrolysates depend on the raw materials, pretreatment methods, and saccharification strategies [24]. Their amounts might directly meet the needs of downstream fermentation production [25,26], be insufficient and require supplementation, or be excessive and have inhibiting effects [27]. Additionally, in some pretreatment and saccharification strategies, especially those whole-cell-based saccharification processes, amino acids, organic acids, vitamins, and other biostimulants might be produced. Although the content of these substances is low, they may have significant impacts on the downstream fermentation process [28].

Nutrients and inhibitory compounds in lignocellulosic hydrolysates are highly dependent on the composition of the raw material, and the methods, strategies, and technologies used in specific pretreatment and saccharification processes [19,29,30,31,32,33,34]. The pretreatment process aims to open the recalcitrant structure of lignocellulose and separate different components as much as possible for subsequent performance [35]. There are many pretreatment methods, including physical (grinding, microwave, ultrasonic, and pyrolysis), chemical (acid, alkali, ozonolysis, organic solvents, and ionic liquids), physicochemical (hot water, steam explosion, ammonia fiber explosion, wet oxidation, and carbon dioxide blasting), biological, and their combinations [36]. While the dense structure of lignocellulose is broken, different pretreatment processes may involve some additional chemical reactions because of the chemical reagents added, high temperature, and high pressure, and some byproducts may be inhibitors for the subsequent saccharification or fermentation [37,38]. For example, some lignin-derived phenolics may be released or converted into more toxic forms during the pretreatment and saccharification, disrupting the integrity of the microbial cell membranes, interacting with or changing the structure of the enzyme’s active sites, thereby inhibiting the enzyme activities [39]. In addition, sugar degradation products (such as furfural) produced during the pretreatment process may directly inhibit enzyme functions and affect the efficiency of the saccharification process [32]. After pretreatment, biomass materials often undergo the washing step for detoxification, but this will significantly increase the cost of pretreatment and the burden of wastewater treatment, so strategies for biomass pretreatment need to be considered comprehensively by combining subsequent saccharification and fermentation processes.

The saccharification process is a key step in biorefinery for producing fermentable sugars. Various saccharification strategies have been developed based on the techno-economic feasibility and difficulty level in implementation. Based on the source of the enzymes used, current saccharification strategies can be divided into off-site and on-site approaches [40]. In the off-site approach, separately produced cellulases are used to convert pretreated lignocellulose into fermentable sugars by enzymatic hydrolysis, such as simultaneous saccharification and fermentation (SSF) which is currently used in the majority of pilot-scale demonstrations and industrial plants. Only enzymes with buffers are usually added in the off-site saccharification approach, and monosaccharides and oligosaccharides are produced, along with other nutrients and inhibitors generated mainly from raw materials and pretreatment processes [41,42]. In the on-site approach, the enzyme production is integrated with the saccharification process in one system, which can significantly reduce the cost of enzyme production and separation, such as consolidated bioprocessing (CBP) and consolidated bio-saccharification (CBS) [40]. Since enzymes are produced directly by microorganisms for saccharification in the on-site approach, metabolic products and partial cell lysates from the enzyme-producing microorganisms are often contained in the resulting lignocellulosic hydrolysate. They can serve as nutrients or inhibitors for downstream fermentation. For example, organic acids may be accumulated by metabolic activities of microorganisms during enzyme production, lead to changes in the pH of the medium, or have toxic effects on the microorganisms, thus affecting the growth of microorganisms and the efficiency of the downstream biorefinery process [39,43,44,45,46].

In summary, lignocellulosic hydrolysates contain carbon sources and other nutrients, as well as various inhibitors. The composition and concentration are closely dependent on the lignocellulosic raw material types, pretreatment processes, and saccharification strategies and processes. This article aims to explore the key stages in the biorefinery process of lignocellulose, summarize how various factors in the biomass, pretreatment, saccharification, and fermentation steps affect nutrient supply and inhibitor formation, and offer new insights for designing lignocellulosic biorefinery processes and improving the efficiency and yield in biorefinery.

## 2. Composition of Lignocellulose Feedstocks

Lignocellulosic biomass contains cellulose, hemicellulose, and lignin as the main components. It also contains small amounts of inorganic elements like potassium, calcium, and magnesium, and various organic compounds such as resins, fats, and waxes that can be extracted with solvents [15,16]. Usually, cellulose and hemicellulose can be hydrolyzed to soluble sugars, which provide carbon and energy sources for microorganisms and are the main nutrients from lignocellulose. Cellulose is a structural homopolymer composed of linear chains constituted by repeating β-D-pyranose glucose units linked by β-(1,4) glycosidic bonds [47]. The cellulose chains are bonded through non-covalent interactions (van der Waals forces and hydrogen bonds), forming rigid and insoluble microfibrils [48]. Depending on the different orientations and different levels of crystallinity, cellulose molecules form amorphous (low-crystallinity) and crystalline (high-crystallinity) regions [49]. A higher crystallinity means that the molecular chains are arranged more tightly and orderly within the crystalline regions, enhancing the material’s mechanical hardness and chemical stability [50,51]. Such complicated structures render cellulose resistant to biological and chemical degradation, making it a major barrier in the conversion process of lignocellulosic biomass.

Hemicelluloses are branched and heterogenic polysaccharides composed of pentose (D-xylose and L-arabinose) and hexose (D-glucose, D-mannose, and D-galactose) [52]. These monosaccharide units are linked by β-(1,4)-glycosidic bonds and β-(1,3)-glycosidic bonds [53]. The amorphous, random structural properties and the lower physical strength make hemicelluloses easier to be hydrolyzed than celluloses, but they can act as a physical barrier for cellulases’ access to celluloses [38]. Therefore, the removal or separation of hemicelluloses is often necessary for the pretreatment process.

Lignin is an amorphous and highly branched phenolic polymer primarily composed of syringyl (S), guaiacyl (G), and p-hydroxyphenyl (H) subunits. Lignin has hydrogen bonding with cellulose and hemicellulose and is also connected to hemicellulose via various alkyl/aromatic ether linkages, forming lignin–carbohydrate complexes (LCCs). These LCCs prevent enzymes from accessing cellulose during enzymatic hydrolysis [13,48] and cause enzyme deactivation by irreversible enzyme adsorption [36]. Therefore, lignin-derived compounds are major inhibitors of enzymatic reactions and microbial fermentation. Moreover, lignin and its derivatives are difficult to be degraded and assimilated by microorganisms. Therefore, it is often necessary to remove lignin and lignin-derived compounds to eliminate their negative effects through pretreatment to obtain more fermentable sugars [53].

The constitution, structure, and distribution of lignocellulose in the cell wall vary significantly from different biomass sources, depending on the plant species, climate conditions, growth stages, and processing methods [54]. For example, cultivars harvested in summer often have a higher cellulose content than those harvested in autumn [55]. The composition of hemicellulose also varies significantly among different plant species and even within different parts of the same plants (such as the leaves, stalks, and roots) [56]. For example, hemicelluloses in softwoods are constituted by glucomannans, arabinoglucuronoxylans (xylans), arabinogalactans, xyloglucans, and other glucans, while, in hardwoods, hemicelluloses are primarily composed of xylans and glucomannans [57]. Generally, the content of hemicellulose in hardwoods and herbages is usually higher than in softwoods [58]. The composition of lignin also varies among plant types. Hardwood lignin primarily contains S and G subunits, with relative amounts of 45–75% and 25–50%, respectively; softwood lignin is mainly composed of G subunits accounting for about 95%; while the relative contents of H/G/S subunits are about 5–35%, 35–80%, and 20–55%, respectively, for herbaceous plants [37,59]. The total lignin contents are also different: softwoods have the highest lignin content, while herbaceous plants have the lowest [60].

Although the components of lignocellulosic biomass are influenced by various factors, their approximate contents in raw materials are relatively stable: 15–30%, 20–40%, and 35–50% for lignin, hemicellulose, and cellulose, respectively [54]. The compositions of some common lignocellulosic biomass are listed in Table 1.

These biochemical characteristics of lignocellulosic components help predict the yield of fermentable sugars and understand the native recalcitrance of raw materials. Generally, the higher the proportion of cellulose in raw materials was, the more glucose could be released; the higher the percentage of lignin present was, the more difficult it was for the biomass to be degraded [64]. Moreover, lignin and hemicellulose, along with their degradation products, can suppress cellulose hydrolysis [17,18]. Therefore, a lignocellulosic biomass with lower contents of lignin and hemicellulose is more suitable for biorefinery.

The different types of lignocellulose can influence the variable amount of inhibitors [29]. For example, more acetylation normally happens in hardwoods than in softwoods [71]. Acetic acid is produced by acetyl-group hydrolysis and has significant inhibiting effects on microbial fermentation. Moreover, acetylation on hemicellulose chains can cause surface hydrophobicity changes, inhibit hydrolases, and contribute to biomass recalcitrance [72,73]. Therefore, the lower the acetylation degree in hemicellulose was, the higher the sugar yield obtained. Due to significant variations of three components in different lignocellulose biomasses, similar pretreatment methods may generate different amounts of inhibitors with diverse inhibiting effects [37]. Major inhibitors derived from lignin are phenolic compounds such as 4-hydroxybenzoic acid, vanillin, catechol, ferulic acid, and syringic acid [19]. Inhibitors derived from hemicellulose include furfural and formic acid as the degradation products of xylose and arabinose. Inhibitors derived from cellulose include 5-hydroxy methylfurfural (5-HMF), formic acid, and levulinic acid [74]. Although the concentration of inhibitors may not be high, their inhibiting effects can be significant [45], and the inhibitory effects of the same inhibitors from different biomass types may be discrepant. For example, the concentration of phenolic compounds released from beechwood ranges between 2–21.6 mg/g after steam explosion pretreatment, and the conversion of cellulose to glucose is reduced by 5–26% [75]. In contrast, the concentration of the phenolic from maple is 5.65 mg/g after liquid hot water (LHW) pretreatment, and the hydrolysis rate is reduced by 50% [76].

In summary, the contents and structural characteristics of cellulose, hemicellulose, and lignin vary among various lignocellulosic materials. This physical and chemical nature of biomass materials affects saccharification yields and inhibitor generation in pretreatment, thereby determining biorefinery strategy selection.

## 3. Pretreatment Process and Its Effects on Nutrients and Inhibitors

Lignocellulose pretreatment is a crucial step for biorefineries, aiming to increase the share of the amorphous region in celluloses, promote hemicellulose degradation, and remove lignins, to enhance the susceptibility of the lignocellulosic biomass to enzymatic degradation [35]. Pretreatment methods are diverse, including acid treatment, alkaline treatment, ionic liquid treatment, deep eutectic solvent (DES) treatment, steam explosion, hydrothermal treatment, physical treatment, and biological treatment, as well as their combinations. When selecting a pretreatment method, it is important to consider the biomass type, the anticipated end products, and the economic benefits [77]. For instance, acid pretreatment is more effective in hemicellulose removal, while alkaline pretreatment is more efficient in delignification [78]. During the delignification and decomposition of hemicellulose, some pretreatment byproducts with fermentation-negative effects are generated due to severe conditions [79]. Qualitative and quantitative assessments of these byproducts are important for determining the suitability of each raw material pretreatment method, including whether hydrolysate detoxification is necessary or not for effective subsequent fermentation.

### 3.1. Alkaline Pretreatment

The following mechanisms for changing the structure and composition of biomass are mainly involved in alkaline pretreatment: first, exposing more celluloses and hemicelluloses by breaking down ether bonds and carbon–carbon bonds between aromatic rings and dissolving lignins; and, second, improving the accessibility of cellulose fibers to enzymes by cellulose swell, surface area increase, and crystallinity reduction. Besides lignin, alkaline pretreatment can partially dissolve hemicelluloses, although it is not as efficient as acid pretreatment, helping more celluloses be exposed to enzymatic hydrolysis and improving the overall conversion efficiency [80,81]. At last, alkaline pretreatment can cleave the ester linkages in lignocelluloses, including bonds between lignin and hemicelluloses, as well as acetyl and other ester groups in hemicelluloses, thus further reducing the degree of polymerization in lignocellulose [82].

Alkaline pretreatment dissolves only a small amount of cellulose and hemicellulose, so nearly the maximum amount of saccharides can be recovered in subsequent steps [30]. It is reported that over 85% of xylan can be extracted from corn stalks under low-alkali-concentration conditions [83]. Common alkaline reagents include NaOH, Ca(OH)_2_, KOH, and ammonia. A high proportion of demethylated phenolics can be produced by alkaline pretreatment using NaOH [77]. Alkaline pretreatment is very effective for lignin removal from softwoods and grasses, and more holocellulose can be kept compared to acid treatment [30]. Overall, alkaline pretreatment can retain more nutrients with the formation of relatively fewer new inhibitors.

However, some issues need to be addressed about alkaline pretreatment, such as the difficult chemical recovery [84], and equipment corrosion which may result in a short lifespan and high maintenance costs. Moreover, compared to other pretreatment methods, alkaline pretreatment may require a longer time to break down the lignin structure, influencing production efficiency [85]. Conditions of high temperature and high pressure also lead to the energy consumption being increased. After alkaline pretreatment, the lignin-rich black liquor generally requires solid–liquid separation before subsequent saccharification and utilization [86]. Inhibitors in the sugar solution, besides unwashed components of black liquor, mainly include lignin fragments deposited on celluloses and LCC (lignin–carbohydrate complexes) released from the fragmented cellulose [87,88]. The effective utilization of the separated lignin is the key to the economic viability of the entire biorefinery process.

### 3.2. Acid Pretreatment

Acid hydrolysis is one of the most common pretreatment methods, mainly relying on inorganic acids (such as sulfuric acid, phosphoric acid, or nitric acid) or organic acids (such as formic acid, maleic acid, or oxalic acid) [61,68,89,90]. Initially, acid molecules cause the breakdown of the glucosidic bonds between cellulose and hemicelluloses, and hydrolyze hemicelluloses partially or completely into monosaccharides or smaller oligosaccharides. Meanwhile, acid pretreatment affects hydrogen bonds between celluloses and hemicelluloses. By altering these hydrogen bonds, acid pretreatment can reduce the crystallinity of celluloses, making them more accessible for enzymatic hydrolysis [82].

However, acidic environments can promote sugar degradation to generate a large number of inhibitors, such as furfural, 5-HMF, and phenolic compounds, affecting subsequent fermentation [91]. In addition to the common inhibitors above, there are other newly discovered substances, such as quinone compounds derived from phenolic compounds, severely inhibiting the growth and fermentability of various typical biorefinery fermentation strains [92]. Typically, the more severe the acid pretreatment is, the more phenolic compounds are generated, especially those with carbonyl groups, as well as acetic acid, furfural, and 5-HMF in the hydrolysate. The concentration of glucose and some oligosaccharides will also decrease due to excessive byproduct conversion when the pretreatment strength is too high [39]. Traditional detoxification methods such as water washing and biological detoxification can remove these inhibitors, but a large amount of fermentable sugars derived from pretreated lignocellulosic biomass are also lost simultaneously. Therefore, biorefinery processes that use acid pretreatment tend to develop methods by improving the inhibitor tolerance of strains for biodetoxification or constructing pathways for quinone biodegradation [93].

In addition, researchers are exploring other alternative acids with less toxicity and easier removal for biomass pretreatment. For example, trifluoroacetic acid (TFA) can obtain soluble sugars such as xylose from hemicelluloses with celluloses undegraded. Due to the easy recyclability of TFA, an additional detoxification stage is not necessary [68]. Levulinic acid (Lev) is another eco-friendly organic acid for pretreatment and can also prevent the lignins from re-polymerization [94]. More than 50% (*w*/*w*) solids are discharged in the dry acid pretreatment system [95] and all inhibitors are retained in the pretreated solids without any wastewater generated. The pretreated solids can be introduced to fungus cultures for biodetoxification, followed by simultaneous saccharification and co-fermentation for ethanol or lactic acid fermentation [96,97,98], significantly reducing wastewater generation during the detoxification process.

### 3.3. Hydrothermal Pretreatment

Hydrothermal pretreatment primarily utilizes H_3_O^+^ ions ionized from water under increased temperature and pressure. These autoionization products act as catalysts during pretreatment. Acetyl groups on xylan chains are cleaved and form acetic acid in the solution to trigger hemicellulose depolymerization, leaving most celluloses and lignins remaining in the pretreated solids [99]. Compared to other chemical pretreatments, hydrothermal pretreatment has the advantage of being eco-friendly and using pressurized hot water as the only solvent [100]. However, like acid pretreatment, it releases or generates several soluble inhibitors such as acetic acid, 5-HMF, and phenolics, along with some sugar degradation byproducts such as furfural and oligosaccharides [54]. These compounds significantly impact the enzymatic hydrolysis efficiency, especially phenolics, whose inhibitory effects cannot be completely relieved even with an increased enzyme load [45]. Studies have shown that 5-HMF and furfural can undergo polymerization or condensation to form pseudo-lignin [101]. These structures tend to deposit as droplets on the surface of the pretreated biomass, reduce the effective contact area for cellulase, and inhibit cellulase activity [102]. Additional steps are required for lignin removal [103], and alkali and ammonium sulfite are currently commonly used to assist in hydrothermal pretreatment for delignification [104,105,106,107,108]. On the other hand, the low separation efficiency of hemicellulose is reported to be a main technical challenge for hydrothermal pretreatment, and various methods and technologies have been studied to improve hemicellulose separation efficiency, such as pH pre-control [109] and metal ion catalysis [110].

### 3.4. High-Pressure Explosion Pretreatment

Steam explosion (SE) is a widely used physicochemical pretreatment method. It treats biomass with high-temperature and high-pressure steam, then rapidly decompresses to break down the lignin–hemicellulose barrier and effectively facilitate subsequent hydrolysis. During the SE pretreatment process, with the temperature increasing, hemicellulose degradation may result from autohydrolysis reactions and inhibitors like furfural and 5-HMF can be generated in side reactions [111,112,113]. Lignin also partially depolymerizes and melts at high temperatures, similar to that during hydrothermal pretreatment, but these dissolved components may be recondensed or transformed afterward [114].

Compared to SE, nitrogen explosion decompression (NED) offers a different pretreatment approach and is particularly suitable for biomass hydrolysis under mild treatment conditions. It operates at lower temperatures, applies a gentler treatment for biomass, and minimizes the generation of inhibitors. It achieves biomass explosion effects through dissolved nitrogen expanding rapidly, breaks down the lignin–hemicellulose matrix to some extent, and promotes hemicellulose dissolution [65]. NED is characterized by its potential environmental friendliness and lower operation temperatures. As an emerging technology, further evaluation is still required for its technological maturity, cost-effectiveness, and adaptability to various biomasses. Additionally, although fewer inhibitors are produced in NED, detailed generation mechanisms and control strategies are still required in further research to ensure the efficiency and reliability of NED in practical applications.

### 3.5. Solvent Pretreament

Organic solvent pretreatment often uses methanol, ethanol, acetone, and organic acids, and removes lignins and some hemicelluloses through solubilization. Compared to other chemical pretreatments, its advantage lies in the ability to recover relatively pure lignin [115]. Sometimes, organic acids, inorganic acids, or alkalis are added as catalysts to lower the operating temperature or increase delignification [116]. This method requires balancing the relationship between the cost and inhibitor generation. Low-boiling-point alcohols are easily recovered but require a high-pressure pretreatment process; acetone, although better at recovering saccharides, has a higher overall cost and is not feasible for large-scale production [117]. Organic acid pretreatment can be carried out under atmospheric pressure but may lead to cellulose acetylation and inhibitory components accumulation in the system. Although most organic reagents used in pretreatment can be recovered by distillation, it is challenging to ensure that they do not remain on the pretreated solids and flow into the downstream sugar solution during large-scale applications.

Compared to organic solvents, deep eutectic solvents (DESs) are stable, biodegradable, and recyclable green solvents [118]. They are composed of different molar ratios of a hydrogen bond acceptor (such as choline chloride) and a hydrogen bond donor (like lactic acid, urea, ethylene glycol, etc.) [119,120]. The DES pretreatment can increase both the digestibility and solubility of lignocellulose and reduce its resistance to enzymatic digestion; it can also selectively break ether bonds to separate lignins and celluloses, thus deconstructing plant cell walls effectively [121]. Additionally, DESs can suppress the re-polymerization of depolymerized lignin and reduce the lignin molecular weight [122]. The DES pretreatment is considered a green and cost-effective process, typically with characteristics of non-toxicity and recyclability [123]. Despite the large number of laboratory studies emerging in recent years, the efficiency, stability, recyclability, and biocompatibility of DES are still the major challenges in large-scale pretreatment under industrial conditions [124,125].

Ionic liquids (ILs) are also known as “green solvents” with the typical constitution of organic cations and organic or inorganic anions. In the lignocellulose biochemical processing, ILs improve the convertibility of biomass and increase the efficiency of enzymatic hydrolysis and fermentation by disrupting non-covalent interactions between lignocellulose components, such as hydrogen bonds between polysaccharide chains, and ether/ester bonds between lignin and carbohydrates [126,127]. Although the formation of inhibitors is diminished, the residual small amounts of ILs still have potential toxicity to enzymes and fermenting microbes [37]. Therefore, it is necessary to use excess water or antisolvent washing after ionic liquid pretreatment to remove ionic liquids, lignin, and other derivatives [126].

Generally, the economic viability of solvent-based pretreatment is determined by the solvent recovery. Most inhibitors in the subsequent enzymatic sugar solution may be from the solvent itself after efficient solvent recovery. The residue solvent affects not only operating costs but also the qualities of the produced sugar solution and other downstream products.

### 3.6. Other Pretreatment Techniques

Physical pretreatment techniques primarily use external mechanical or electrical forces, such as milling, ball milling, extrusion, ultrasonication, and microwave irradiation [128,129]. The mechanical pretreatments disrupt the intrinsic structure of biomass, thereby increasing the surface area, reducing crystallinity, and improving efficiency. However, mechanical forces cannot break down the chemical structures of lignins and have little effect on the degradation of hemicellulose and lignin. Ultrasonication pretreatment is based on the cavitation effect during radiation with ultrasonic energy, which produces both physical forces and chemical effects on the biomass structure. Microwave irradiation provides rapid and uniform heating effects on the lignocellulose, thus generating structural changes in biomass. However, both ultrasonication and microwave irradiation pretreatments have a high energy demand and are difficult to scale up for high-volume applications. Physical pretreatment is often used in conjunction with other pretreatment methods [128,130,131], but new sources of inhibitors are also introduced. Biological pretreatment is mainly carried out by microorganisms utilizing biomass for growth directly or by enzyme mixtures added. No inhibitors form and less energy is consumed during biological pretreatment [82]. However, compared to chemical pretreatment, it takes longer and has limited effects on facilitating subsequent saccharification.

Although numerous pretreatment methods have been developed, none of them can perfectly achieve the separation of the three major components of lignocellulose. Other compounds, such as extractives and ash, are largely lost during pretreatment. The choice of pretreatment process primarily affects the carbon sources for downstream bioconversion and inhibitor formation. Besides the total process cost, there are two more factors highly recommended for pretreatment evaluation: accelerating polysaccharides hydrolysis or not, and reducing side reactions or not for more main carbon sources remaining and fewer inhibitors generated. Generally, more control over inhibitor generation is required during acid pretreatment, while chemical reagent recovery and lignin utilization need to be addressed in the methods based on alkalis and solvents that focus on delignification.

The possible inhibitors from different pretreatments are summarized in Table 2.

## 4. Saccharification Process

Saccharification is a key step in the biotransformation process of lignocellulose, aiming at breaking down complex polysaccharides of cellulose and hemicellulose into fermentable oligosaccharides and monosaccharides through enzymatic hydrolysis [12]. These oligosaccharides and monosaccharides are primary nutrients for subsequent microbial fermentation, providing carbon sources and energy for the production of ethanol, biofuels, or other chemicals [40,136]. The saccharification process is mainly catalyzed by various enzymes, and the efficiency and yield of saccharification are two of the most critical factors for determining the utilization rate of lignocellulosic polysaccharides. The saccharification process involves the synergistic action of multiple enzymes, primarily glycoside hydrolases (GHs) including cellulases and hemicellulases [57,137,138]. The cellulase system primarily includes the following three types of enzymes: endoglucanases (EGs), which break down the crystalline structure of cellulose microfibrils to release individual polysaccharide chains and reduce the polymerization degree [57]; exoglucanases, or cellobiohydrolases (CBHs), that cut cellulose from the free ends of polysaccharides, mainly releasing cellobiose; and β-glucosidases, which can further hydrolyze cellobiose and other oligosaccharides to produce individual glucose molecules. These enzymes work together to break down celluloses into glucose units [12]. Unlike celluloses, the structure of hemicelluloses is very complex and requires a wider variety of enzymes for complete degradation, mainly including xylanases, arabinoxylanases, mannanases, β-xylosidases, and esterases [12,138]. These enzymes work synergistically and degrade polysaccharides specifically according to the chemical composition and different linkages in hemicelluloses, releasing sugar monomers such as xyloses and mannoses [13]. The efficiency of these enzymes is influenced by various factors, including the various biomass sources, the chemical composition after biomass pretreatment, the source of enzymes, the ratios of different enzymes, and the enzyme catalytic activities [13,139,140]. In addition to glycoside hydrolases, recent studies discovered that adding auxiliary enzymes such as lytic polysaccharide monooxygenases (LPMOs) during the cellulose hydrolysis process can significantly improve hydrolysis efficiency and reduce the enzyme loadings required for saccharification [141,142,143,144].

**Figure 2:**
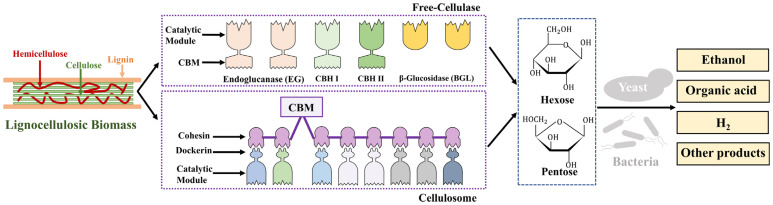
Free-cellulase- and -cellulosome-based saccharification.

Current enzyme systems for biomass saccharification primarily include free enzyme systems and cellulosome systems (Figure 2). Most lignocellulose-degrading bacteria and fungi in nature can secrete a variety of cellulases and hemicellulases. Some fungi, such as *Trichoderma reesei* and *Penicillium oxalicum*, possess a high cellulase secretion system and have been modified to become main production strains of cellulases for industrial-scale use [145,146,147]. Due to the complex structure of lignocellulose, currently, no microbial enzyme can independently decompose all components for industrial application, so many studies are focusing on designing optimal enzyme mixtures to hydrolyze the pretreated biomass effectively [13]. Most industrial demonstration plants for biomass saccharification currently use processes based on free enzyme formulation [148,149,150,151]. Despite the crucial role and multiple advantages of these free enzymes, such as the specific activity, mild reaction conditions, and environmental friendliness, the high enzyme usage in the saccharification process, even with recent significant improvements in enzyme production, still represents a substantial cost in the biorefinery process. This enzyme cost is one of the main factors hindering the economic viability of biorefinery processes [152,153].

In addition to free enzymes produced by fungi and bacteria [154], there exists in nature a large multi-enzyme complex produced by anaerobic microorganisms for degrading lignocellulose, known as cellulosome, composed of non-catalytic proteins (scaffoldins) and a variety of glycoside hydrolases [155,156]. The cellulosome is one of the most efficient lignocellulose degradation systems known in nature, with a far higher degradation efficiency than that of free cellulase systems [154,157,158,159]. The cellulosome assembles various types of cellulases and hemicellulases into a large complex through non-covalent interactions between scaffoldins and enzymes [160,161], creating synergistic and proximity effects among enzymes. Cellulosome also binds to substrates through carbohydrate-binding modules (CBMs), forming synergistic enzyme-substrate interactions. Furthermore, the cellulosome attaches to the bacterial cell wall through cell-wall-binding modules on scaffoldins, creating synergistic interactions between the enzymes and cells [154,158,162]. These multi-level synergistic actions collectively enhance the efficiency of lignocellulose degradation. The modules within the cellulosome are connected by flexible linkers, allowing the cellulosome to undergo conformational changes according to the substrate, thus degrading the substrate better [162]. Additionally, bacteria express genes of cellulosomal components dynamically based on the type of substrate and adapt to the different substrate compositions [162,163,164,165,166]. Compared to free cellulases, cellulosomes not only exhibit a stronger tolerance to chemical inhibitors such as formate, lactate, and furfural present in the hydrolysate but also show a higher ethanol tolerance and thermostability [157].

The biocatalytic saccharification process can be inhibited by various factors, and the most common one is product inhibition, which is that the accumulation of product sugars inhibits enzyme activity. For instance, cellobiose and cello-oligosaccharide inhibit the activity of various cellulases, and β-glucosidases are susceptible to glucose inhibition [167,168]. Typically, the cellobiose inhibition of cellulases is more severe than the glucose inhibition of β-glucosidases. To relieve feedback inhibition and promote cellulose saccharification, strategies often applied are the direct supplementation of exogenous β-glucosidases with a high glucose tolerance or using recombinant strains secreting these enzymes [169,170]. Cellobiose and xylan also inhibit cellobiohydrolases and cellulases, respectively. Notably, studies have found that, under certain conditions, xylose at low concentrations can stimulate β-glucosidases with doubled hydrolytic activity, while the binding of cellobiose to the active site of the enzyme may be interfered by high xylose concentrations [171]. This phenomenon implies the need for precise control over the effects of different components on enzyme activity for saccharification condition optimization and efficiency improvement. In the cellulosome system, it was found that soluble lignin and arabinoxylan released during lignocellulose hydrolysis can interact with key exoglucanases [137,172]. In biorefinery strategies that integrate the saccharification process with downstream fermentation (such as simultaneous saccharification and fermentation or consolidated bioprocessing), the products of downstream fermentation, such as bioethanol, can significantly inhibit cellulase activity as their concentration increases, sometimes even leading to enzyme deactivation [173].

Since the saccharification process is the core step to generate nutrients for downstream fermentation in lignocellulose biorefining, various current lignocellulose bioconversion strategies have been developed based on biocatalyst production methods and their integration with upstream and downstream processes, including off-site and on-site saccharification [40]. Off-site saccharification strategies are among the earliest proposed biorefining technologies, and separate hydrolysis and fermentation (SHF) and simultaneous saccharification and fermentation (SSF) are the most commonly applied, both of which use free cellulases from fungi as biocatalysts. Considering the joint utilization of pentose and hexose downstream, separate hydrolysis co-fermentation (SHCF) and simultaneous saccharification co-fermentation (SSCF) strategies have been further developed [174]. On-site saccharification strategies are new approaches developed for their low operating cost, especially the enzyme production cost by avoiding enzyme production and separation and integrating the enzyme production and saccharification steps into one single step, mainly including consolidated bioprocessing (CBP) and consolidated bio-saccharification (CBS) [40]. On-site saccharification requires a single enzyme-producing strain to produce all enzymes needed for saccharification, thus demanding a higher capacity of the strain for lignocellulose degradation. In addition, the tolerance of the strain is also required to be higher because of the multi-step integration.

The lignocellulosic hydrolysates produced by on-site and off-site strategies are quite different for downstream fermentation. In off-site saccharification, free enzymes are used as catalysts for the saccharification process, so the nutrients and inhibitors in the hydrolysates mostly come from the lignocellulosic substrates themselves and pretreatment processes, while, in on-site saccharification, the hydrolysates are also a fermentation medium for enzyme-producing strains [40]; therefore, besides the nutrients and inhibitors from the substrates and pretreatment processes, metabolic products from the strains are also contained in hydrolysates, and both nutrients and potential inhibitors may be included for downstream fermentation [3]. The differences among lignocellulose hydrolysis in various saccharification approaches will be further discussed in the next section.

## 5. Lignocellulose Hydrolysate in Different Biorefinery Strategies

**Figure 3:**
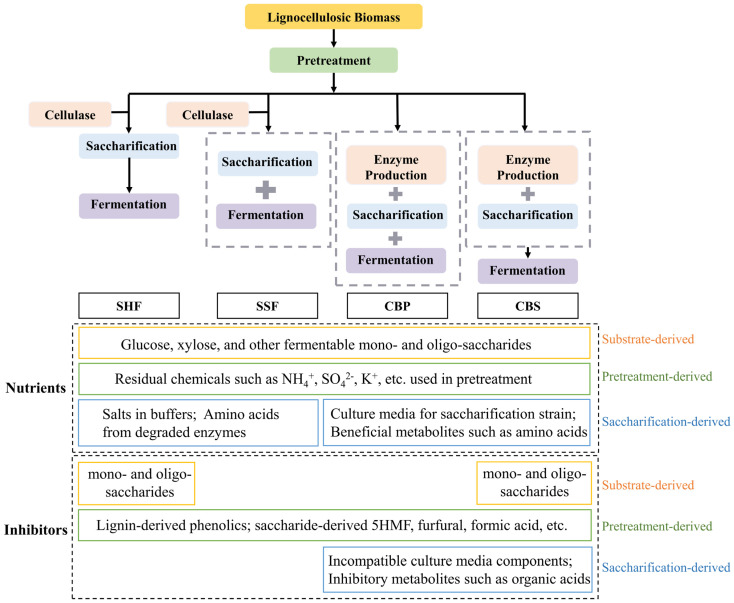
Nutrients and inhibitors in different biorefining strategies. SHF: separate hydrolysis and fermentation; SSF: simultaneous saccharification and fermentation; CBP: consolidated bioprocessing; and CBS: consolidated bio-saccharification.

The production cost of biofuels or fermentable sugars as intermediate platform products from biorefinery requires competitiveness with fossil fuels or starch-based sugars on the market. Therefore, reducing the operational cost of biorefining is the primary concern for strategy development. To overcome the techno-economic challenges of biorefinery, several different strategies have been developed, including separated hydrolysis and fermentation (SHF), simultaneous saccharification and fermentation (SSF), consolidated bioprocessing (CBP), and consolidated bio-saccharification (CBS) [139]. These strategies, by separating or combining different steps of biorefinery, result in variations of obtained lignocellulosic hydrolysates (Figure 3). Such differences further affect the performance of downstream fermentation, which is a critical factor that needs to be considered in the designs of downstream fermentation products and processes.

SHF is a strategy in which each step—pretreatment, enzyme production, saccharification, and fermentation—is carried out separately. The advantage of SHF is that the saccharification and fermentation processes can be conducted under their optimal conditions respectively, thus achieving higher yields [175]. However, the main disadvantage is that each step is performed separately, so a high overall process cost is generated owing to specific operational costs for each step, and additional costs are also introduced by the connections between steps. The lignocellulosic hydrolysate produced by SHF, due to the precise control over each step, typically contains high concentrations of fermentable sugars [176,177]. The inhibitors for fermentation primarily originate from raw materials and the pretreatment process, allowing the generation of nutrients and inhibitors to be better controlled. High sugar yields can be achieved in SHF, with most of the pentoses from hemicellulose retained in the fermentable sugars after saccharification. Therefore, the main consideration for downstream fermentation in SHF is the joint utilization of pentoses and hexoses for the polysaccharide nutrients in lignocelluloses to be fully converted and utilized [42]. The strategy that enables the joint utilization of pentoses and hexoses in SHF is also known as separate hydrolysis co-fermentation (SHCF) [178].

SSF integrates saccharification and fermentation simultaneously in a single bioreactor, reducing operating costs while avoiding the accumulation of high concentrations of sugars in the hydrolysate and related inhibitory effects. However, the optimal conditions for saccharification and fermentation are usually different, especially those of temperature and pH. The optimum temperature for enzymatic hydrolysis is usually higher than that for fermentation, thus typically leading to reduced overall efficiency. Meanwhile, metabolites generated during the fermentation process may inhibit enzyme activities in the saccharification process [31]. To address these issues, many works of research are focusing on enzyme optimization and fermentative microorganism screening to ensure both of them can work efficiently under the same conditions [179,180,181,182,183]. Co-cultures of multiple microorganism strains are also often applied in SSF [184,185,186]. This strategy is advantageous for more a complex metabolite production and is beneficial for strain growth and fermentation processes due to the synergistic interactions between different strains [28]. However, some studies have also shown that the co-culture strategy in SSF may lead to reduced yields due to various reasons (such as nutrient competition and the accumulation of metabolic products) [27].

In SSF, due to the fermentation step integrated, small molecule sugars produced from the hydrolysis of celluloses and hemicelluloses are directly utilized by fermentation strains; thus, lignocellulosic hydrolysates cannot be generated with high sugar concentrations. However, like SHF, SSF also faces issues of inhibitor production during the pretreatment process and joint utilization of pentoses and hexoses. Regarding inhibitors, some studies focus on improving pretreatment methods or using inhibitor-resistant or inhibitor-utilizing strains [187]. Other processes such as simultaneous saccharification and co-fermentation (SSCF) have been developed based on the SSF to achieve effective xylose utilization and less product inhibition [10,188,189]. The approach known as fed-batch delayed SSF (fed-batch dSSF) has also been developed: dSSF (also known as PSSF, pre-hydrolysis SSF [190]) is performed in the first bioreactor by using cellulases for pre-saccharification and followed by simultaneous saccharification and fermentation, while, in the second bioreactor, a cultivation medium was used only for enzymatic hydrolysis. When glucose is depleted in the first bioreactor, the medium is fed from the second bioreactor. This process is primarily designed to avoid early carbon deficiency in SSF and to enhance the hydrolysis rate of cellulase into glucose by integrating a pre-saccharification step at the optimum temperature for cellulose decomposition [41].

Consolidated bioprocessing (CBP) integrates the enzyme production, saccharification, and fermentation steps into a single system, thus significantly reducing the costs of enzymes and the overall process. The core of CBP is to develop microorganisms with the capability of degrading lignocellulose and conducting downstream fermentation simultaneously [191]. Similar to SSF, hydrolysates with high sugar concentrations do not exist in CBP, but issues about inhibitors produced from substrates and the pretreatment process, as well as inhibitory effects of fermentation products on microorganisms and enzymes, have to be faced in CBP. The whole CBP process is carried out by living microbial cells, and the derived inhibitors from pretreatment and raw materials may interfere with the host cell membrane integrity, protein synthesis, cell growth, and target product production [32]. In the CBP system, since all steps occur in the same bioreactor, the efficient consumption of the sugar mixture or the biomass hydrolysate is particularly critical. For instance, the hydrolysis of hemicelluloses produces xyloses, arabinoses, galactoses, and rhamnoses, but many host microorganisms in CBP cannot consume these sugars, leading to a carbon catabolite repression [192]. In such cases, CBP hosts are required to be modified by genetic engineering or metabolic engineering [32,193,194]. Another approach is the co-culture strategy by introducing other strains to metabolize these sugars and fully utilizing various carbohydrates in the lignocellulosic hydrolysates, thus improving the hydrolysis efficiency of upstream strains [191,195,196]. However, this approach has its challenges, as the growth conditions in the co-culture system should meet the requirements for all different microorganisms (such as pH, oxygen, and temperature) and the growth of one species does not have toxic or inhibitory effects on others [197]. In lignocellulose biorefinery, most cellulases exhibit optimal enzymatic activity at higher temperatures; thus, downstream strains are required to adapt to such a high-temperature environment for growth and fermentation. Cellulosomes of *Clostridium thermocellum* exhibit a higher efficiency in degrading woody and herbaceous cellulose materials than commercial fungal cellulases [198], although their fermenting property is unsatisfactory. However, the co-culture of *Clostridium thermocellum* DSM1313 and *Thermoanaerobacterium thermosaccharolyticum* MJ1 can produce more hydrogen [196,199]. This is due to relieving the inhibition of DSM1313, improving substrate degradation, and enhancing electron transfer activity. Additionally, besides substrate utilization, the co-culture system can also improve the low tolerance of organic acids (such as acetic acid, formic acid, and lactic acid) and ethanol produced during fermentation with *Clostridium thermocellum* [33,34]. Overall, more complex modifications of strains are required in the CBP strategy to make full use of hydrolysis products and tolerate various inhibitors produced during pretreatment and fermentation.

Consolidated bio-saccharification (CBS) is a strategy that separates the fermentation step in CBP while keeping the enzyme production and saccharification process completed by a single strain [40]. The key advantage of the CBS strategy is that it integrates enzyme production with saccharification to minimize the production cost of cellulases, overcoming the major bottleneck in lignocellulosic biorefinery. Furthermore, it also separates the cellulose hydrolysis process from the downstream microbial fermentation process, avoiding the compromise of different reaction conditions required in both processes. Since the fermentation process is separated, the CBS strategy, like the SHF strategy, can produce hydrolysates containing high concentrations of fermentable sugars, while cellulases may suffer from the feedback inhibition of monosaccharides or oligosaccharides in the hydrolysates. Therefore, as in the SHF strategy, the product feedback inhibition by cellobiose and cello-oligosaccharide can be released by the addition of β-glucosidases (BGLs) with both a high enzymatic activity and high glucose tolerance in the CBS strategy [170]. However, whole-cell catalysts are used for the saccharification process in the CBS strategy, which is different from the SHF strategy, so the resulted lignocellulosic hydrolysates are also the fermentation broth of the CBS strain, containing more complex components than those of the SHF strategy. These components mainly come from the fermentation medium and bacterial metabolites, so most of them may act as nutrients for downstream fermentation and may be beneficial for the downstream fermentation process. For example, Liu et al. [26] successfully achieved the fermentation of pullulan using condensed CBS sugar liquor without any nutrient supplementation. Similarly, lactic acids were produced by directly inoculating lactic acid bacteria (LAB) strains into the CBS hydrolysates to initiate the fermentation process [3]. On the other hand, since the strains used in CBS are anaerobic *Clostridium thermocellum* [200], certain amounts of metabolic intermediates such as lactic acid and acetic acid are produced, which may inhibit some anaerobic fermentation processes for bioenergy production, such as acetone–butanol–ethanol (ABE) fermentation [157,201]. Therefore, lignocellulosic hydrolysates in the CBS strategy require further analysis of their components, integration with downstream fermentation processes, nutrients needed for downstream fermentation, and potential inhibitors, thereby optimizing the whole CBS process and downstream fermentation processes for the best-matched optimal process.

In this section, we have analyzed the advantages and limitations of various biorefining strategies in terms of nutrient retention and inhibitor control. Overall, SHF allows enzyme production, saccharification, and fermentation under optimal conditions, which can significantly preserve the nutrients from lignocellulose (sugar yield) and independently optimize each step for minimizing inhibitor accumulation or residues. However, the operational cost is high, making techno-economic viability the primary challenge. The SSF strategy effectively integrates the saccharification and fermentation processes, reducing process costs, but it also faces challenges such as high enzyme costs and difficulties in matching saccharification with fermentation. The CBP strategy, theoretically, can achieve lower costs but has extremely high requirements for microbial strains, and there is still a long way to go to develop technologically and economically viable CBP strains. The CBS strategy combines the low-cost advantage of CBP with the high yield of SHF, but the produced saccharification liquid has complex components, and it is necessary to develop compatible downstream strains.

The overall consideration of the biorefinery strategy and process to maximize nutrient retention and control inhibitors at a low cost is critical for the techno-economic assessment (TEA) and the life cycle assessment (LCA) of different biorefinery scenarios [202,203,204,205]. Due to the long research history and the establishment of many demonstration pilot plants of off-site biorefinery approaches, there are many TEA and LCA analyses of SHF and SSF strategies, as well as the specific steps of them such as feedstocks and pretreatments [204,206,207,208,209,210,211,212,213]. The TEA and LCA analyses of on-site approaches are relatively less, but limited studies have shown that the CBP strategy has significant advantages in feasibility and sustainability compared with SHF/SSF strategies [214,215,216]. As we analyzed in this paper, both nutrients and inhibitors will run through the entire biorefinery process, so any improvements to a single step will require a further TEA and LCA analysis of the entire biorefinery, which needs to be greatly strengthened in future research.

## 6. Conclusions and Perspective

This article provides an in-depth analysis of how different steps in lignocellulose biotransformation affect the nutrients and inhibitors in lignocellulosic hydrolysates, highlighting the differences in raw material selection, pretreatment methods, and biorefinery strategies in terms of nutrient production and inhibitor control. We found that, although each strategy has its unique advantages, it also faces various challenges such as cost, efficiency, inhibitory product generation, and difficulties in strain development. A vast amount of research focuses on nutrient retention (increasing fermentable sugar yields or conversion rate), the control or removal of inhibitors, and strain development for specific strategies, but there are only limited studies that considered the overall compatibility of steps in the entire lignocellulosic bioconversion process. Our analysis shows that both nutrients and inhibitors can be produced throughout all biorefinery stages, and, at the same time, each subsequent step is closely related to the nutrient retention and inhibitor generation in the previous step. To achieve the techno-economic viability of lignocellulosic biorefining, it is necessary to consider all steps comprehensively, design the most suitable biorefining strategy based on the characteristics of the raw materials and final target products, and optimize the whole process to maximize nutrient retention and conversion and minimize inhibitor generation.

Therefore, we propose that future research on lignocellulosic biorefinery should focus more on matching the overall biorefinery strategy and process, especially the compatibility of the pretreatment, enzyme production, and fermentation strain development, to maximize nutrient retention and control inhibitors at a lower cost. For example, pretreatment technologies should be developed based on the requirements of downstream saccharification and fermentation processes. It is necessary to analyze the role of residual chemicals or byproducts during pretreatment more meticulously and thoroughly because they can be inhibitors for subsequent saccharification and fermentation, act as activators for enzyme activity, or provide nutrients for fermentation. For instance, nitrogen sources required for the growth of downstream fermentation microorganisms often need to be added additionally. The ammonium salts, such as ammonia or ammonium sulfite, from nitrogen-containing pretreatment processes, may move into the microbial conversion stage with residual lignin, but their stimulative or inhibitory effects on fermentation still require further studies. Most previous research on nutrient retention, inhibitor control, or removal focused on off-site strategies (SHF and SSF), while very limited studies target more recent on-site strategies (CBP and CBS). *Clostridium thermocellum* is currently used in on-site strategies, whose core of the saccharification process is their secreted cellulosomes. However, research about the effects of various sugars, residues, and byproducts from pretreatment on the activity and stability of *Clostridium thermocellum* with their cellulosomes is still very few and should be focused on in the future. For the CBS strategy first developed in our lab, we are committed to the investigation of the overall compatibility of steps in the entire lignocellulosic bioconversion process. For example, the composition of the produced saccharification liquid is complex, and it should be developed in collaboration with downstream processes. The medium used in the CBS process for enzyme production and saccharification contains various nutrients required by *Clostridium thermocellum* growing, and most of them can remain in the final saccharification liquid in various forms, along with some products generated from *Clostridium thermocellum* metabolism. More detailed studies need to be carried out about the suitability between these components and downstream fermentation strains, which are ongoing in our lab for the CBS strategy. Moreover, the salts in the CBS hydrolysates come from not only the medium but also ash in the substrate dissolved during pretreatment and saccharification. These salts have potentially significant effects on downstream fermentation equipment and processes. The choice of medium in an on-site saccharification strategy not only determines the production status of the enzyme-producing microorganisms but also affects crucial carbon source supply. Moreover, the inhibitory effects of metabolites and the changes in nutrient components during the enzyme production process of the on-site saccharification strategy also require an overall assessment of their impacts on the performance of production strains. More TEA and LCA studies for different biorefinery approaches, particularly the more recent on-site strategies (CBP and CBS), with improved nutrient retention and inhibitor control are needed to validate their feasibility and sustainability.

## Figures and Tables

**Table 1:** Content of cellulose, hemicellulose, and lignin in common lignocellulosic biomass.

Biomass	Cellulose (%)	Hemicellulose (%)	Lignin (%)	Reference
Sugarcane bagasse	32–55	22–36	14–30	[61]
Sugarcane straw	29	28.8	32.2	[45]
Sorghum straw	26.93	32.57	10.16	[62]
Wheat straw	43.4	26.9	22.2	[63]
Barley straw	35.73–45.73	26.8–32.6	5.3–5.9	[64,65]
Aspen wood	50.7	16.6	13.3	[64]
Oak	43.2	21.9	35.4	[66]
Corn stover	38	23	20	[67]
Switchgrass	50	40	20	[67]
Pine chip	33–44.78	17.56–23.75	20.22–26.29	[68,69]
Spruce	24.7	10.2	35	[70]

**Table 2:** Summary of nutrient retention and inhibitor formation under various pretreatment methods.

Pretreatment Method	Nutrient Retention	Inhibitor Production	Reference
Alkaline pretreatment	Removal of lignin, partial hemicellulose; less sugar dissolution	Formic acid; acetic acid; hydroxy acid; phenols	[37,77]
Acid pretreatment	Partial or complete removal of hemicellulose; more sugar dissolution	Furfural; 5-HMF; phenols; quinones; acetic acid	[91,92]
Steam explosion	Significant dissolution of hemicellulose, minor dissolution of cellulose; less degradation of sugar	Furfural; 5-HMF; formic acid; acetic acid	[36,111,112,113]
Nitrogen explosion	Hemicellulose dissolution		[65]
Liquid hot water	More hemicellulose dissolved; higher sugar recovery; less cellulose loss	Furfural; 5-HMF; acetic acid; phenols; pseudo-lignin	[2,36,54]
Organic solvent	Removal of part of the hemicellulose, dissolution of lignin	Furfural; 5-HMF	[132]
Deep eutectic solvents	Removal of hemicellulose and lignin	Furfural; 5-HMF; levulinic acid	[123]
Ionic liquid	High lignin extraction rate, partial degradation of hemicellulose, possibly reduced cellulose crystallinity	Furfural; 5-HMF; weak acid	[37,126,133,134]
Physical pretreatment	Reduced cellulose crystallinity, less sugar degradation	Furfural; phenols	[36,133,135]
Biological pretreatment	High lignin degradation, low cellulose degradation, reduced sugar	Furfural; 5-HMF; organic acids	[36,133]

## Data Availability

No new data were created or analyzed in this study. Data sharing is not applicable to this article.
